# A Trajectory Collaboration Based Map Matching Approach for Low-Sampling-Rate GPS Trajectories

**DOI:** 10.3390/s20072057

**Published:** 2020-04-06

**Authors:** Wentao Bian, Ge Cui, Xin Wang

**Affiliations:** 1 School of Information Science and Technology, Northwest University, Xi’an 710127, China; wtbian@stumail.nwu.edu.cn (W.B.); xcwang@ucalgary.ca (X.W.); 2 Intelligent Transportation Systems Research Center, Wuhan University of Technology, Wuhan 430070, China; 3 Department of Geomatics Engineering, University of Calgary, Calgary, AB T2N1N4, Canada

**Keywords:** map matching, low-sampling-rate GPS trajectories, trajectory collaboration, trajectory clustering

## Abstract

GPS (Global Positioning System) trajectories with low sampling rates are prevalent in many applications. However, current map matching methods do not perform well for low-sampling-rate GPS trajectories due to the large uncertainty between consecutive GPS points. In this paper, a collaborative map matching method (CMM) is proposed for low-sampling-rate GPS trajectories. CMM processes GPS trajectories in batches. First, it groups similar GPS trajectories into clusters and then supplements the missing information by resampling. A collaborative GPS trajectory is then extracted for each cluster and matched to the road network, based on longest common subsequence (LCSS) distance. Experiments are conducted on a real GPS trajectory dataset and a simulated GPS trajectory dataset. The results show that the proposed CMM outperforms the baseline methods in both, effectiveness and efficiency.

## 1. Introduction

With the development of positioning technology, massive GPS trajectory data has been continuously generated from vehicles such as cars, taxis and buses. A GPS trajectory is a sequence of GPS points which records the spatial track of a moving object. As a GPS trajectory can deviate from its actual location in the road network, caused by device malfunction, urban canyons and other positioning errors, many map matching methods have been proposed to locate GPS trajectories onto road networks. The core problem of map matching is the uncertainty issue of GPS trajectories, including the uncertain path between consecutive GPS points, and this problem becomes more severe for low-sampling-rate GPS trajectories.

**Figure 1:**
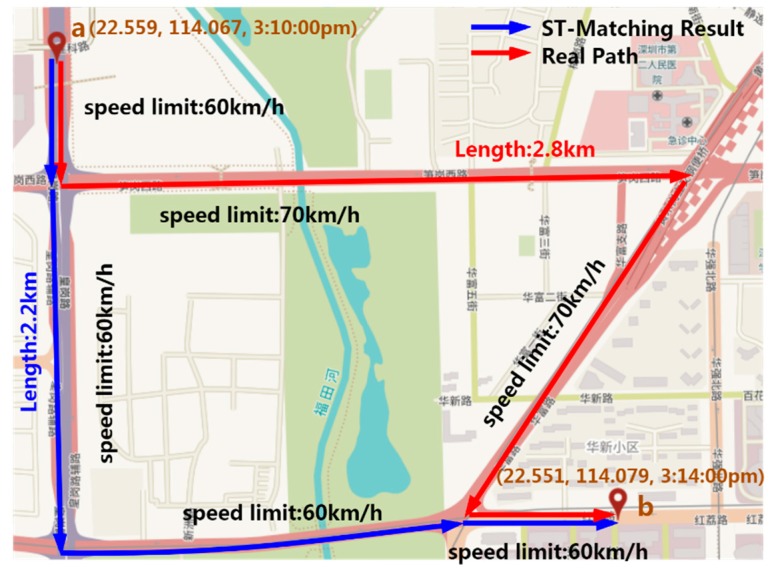
Example of map matching result based on spatial and temporal model for a low-sampling-rate GPS trajectory.

At present, GPS trajectories with low sampling rates (the time interval between consecutive GPS readings exceeds 1 min) are collected for many applications as this can conserve battery life and help track vehicles for a long period. The current map matching algorithms are developed to determine the correct path of low-sampling-rate GPS trajectories, based on various features, such as spatial [1,2], temporal [2], speed constraint [3,4] and turning information [5]. However, these algorithms do not perform well when sampling rates are very low, i.e., the sampling rate exceeds three or four minutes, especially in a dense road network. Spatial features (e.g., distance similarity) and temporal features (e.g., speed similarity) [2,6] are sometimes ineffective in identifying the correct path between consecutive GPS points, when they are far from each other. Figure 1 shows an example of the map matching process based on spatial and temporal features. In the figure, point **a** (22.559° N, 114.067° E, 3:10:00 p.m.) and point **b** (22.551° N, 114.079° E, 3:14:00 p.m.) are two consecutive GPS points of a low-sampling-rate trajectory. Two candidate paths (shown in blue and red) are identified with the lengths of 2.2 km and 2.8 km, and the average speeds moving along the two paths between **a** and **b** are 33 km/h and 42 km/h, respectively. As shown in the figure, the speed limits of road segments on the blue path are (60 km/h, 60 km/h, 60 km/h and 60 km/h), and on the red path are (60 km/h, 70 km/h, 70 km/h and 60 km/h). According to the spatial and temporal similarities of the ST-Matching algorithm [2], the blue path is more likely to be the map matching result than the red path, since the length of the blue path is shorter than that of the red path. Additionally, the cosine similarity value between average speed and the speed limits of road segments on the blue path is larger than the cosine similarity value on the red path. However, the red path is the actual path taken by the user. The example shows that using only spatial and temporal features sometimes does not effectively reduce the large uncertainty and distinguish the correct path for GPS trajectories with very low sampling rates.

Other methods attempt to reduce the uncertainty of low-sampling-rate GPS trajectories with the addition of historical high-sampling-rate GPS trajectories. For instance, historical high-sampling-rate GPS trajectories are used to detect frequent path patterns, and the path of a low-sampling-rate GPS trajectory is estimated based on the discovered patterns [7,8]. However, historical high-sampling-rate GPS trajectories are not always available. Furthermore, the quality of these map matching methods heavily relies on the effectiveness of the discovered patterns. With rapid road development or traffic control, the frequent path patterns, discovered from historical GPS trajectory data, may not be applicable for the current situation. Therefore, creating effective map matching for low-sampling-rate GPS trajectories without auxiliary data continues to be a challenging problem.

This paper intends to solve the uncertainty issue of low-sampling-rate GPS trajectories through trajectory collaboration. Similar low-sampling-rate GPS trajectories are aggregated into clusters, and a collaborative GPS trajectory is then generated for map matching from each cluster with information supplementation. However, this method presents several challenges.

First, an effective clustering algorithm for low-sampling-rate GPS trajectories is significant. There are many methods of trajectory clustering, such as the partition and group method [9], trajectory clustering with Fréchet distance [10] and clustering algorithm for network constraint trajectories (NETSCAN) [11]. These algorithms are designed for different applications. For example, the clustering algorithm in [9] is designed for flock or typhoon trajectories, but does not consider road constraint in map matching. The clustering algorithms in [10,11] consider road constraint and calculate the geometric similarity between GPS points, but they are not applicable for low-sampling-rate trajectories. The reason for this is that the distance between consecutive GPS points is large (maybe larger than 500 m) in low-sampling-rate GPS trajectories, so that the geometric similarity between trajectories would be small, even on the same path. Exploring an effective method of clustering low-sampling-rate GPS trajectories is worth studying.

Second, extracting useful information from a group of similar GPS trajectories to reduce the path uncertainty issue is required. After trajectory clustering, low-sampling-rate GPS trajectories in each cluster are still separated from each other and are not collaborated for map matching. An issue remains understanding how to use a group of similar GPS trajectories to reduce uncertainty by trajectory collaboration and further benefit map matching. 

In this paper, a collaborative map matching algorithm called CMM is proposed to address low-sampling-rate GPS trajectories. CMM processes a group of similar GPS trajectories together, rather than as individual trajectories, which makes map matching more efficient. In the preprocessing step, CMM removes outliers from low-sampling-rate GPS trajectories. Spatial indices, including an R-tree and a Path-Forest, are built to facilitate candidate path search. Then, a DBSCAN (Density-Based Spatial Clustering of Applications with Noise) algorithm is extended to cluster GPS trajectories into different groups, based on path similarity instead of geometric similarity. Path similarity is designed for similarity measurement between low-sampling-rate GPS trajectories by comparing their candidate paths. Next, a resampling method is applied to generate a new collaborative GPS trajectory from each trajectory cluster. Finally, the generated collaborative GPS trajectory is matched to the path candidate with the highest LCSS (Longest Common Subsequence) similarity.

The specific contributions of this research are highlighted below:

1. This paper proposes a collaborative map matching algorithm called CMM to address low-sampling-rate GPS trajectories based on trajectory clustering and resampling. CMM does not rely on any additional information, such as historical high-sampling-rate GPS trajectories or traffic flow information.
2. DBSCAN clustering algorithm is extended to aggregate similar low-sampling-rate GPS trajectories together, based on path similarity, which can effectively decrease the negative impact of geometric similarity on clustering of low-sampling-rate GPS trajectories.
3. A resampling method, based on a sliding window, is proposed to generate a new collaborative GPS trajectory from each trajectory cluster. The generated collaborative GPS trajectory integrates the collective information of similar low-sampling-rate GPS trajectories.
4. Experiments have been conducted based on a simulated GPS trajectory dataset, and the results show that the proposed CMM algorithm outperforms the baseline methods in both, effectiveness and efficiency.

This paper is organized as follows: Section 2 introduces related works on map matching. Section 3 provides a detailed discussion on the proposed CMM algorithm. Section 4 discusses experiments based on both a real and a simulated GPS trajectory dataset. Section 5 presents the conclusions and discusses future works resulting from this research.

## 2. Related Work

### 2.1. Map Matching Problem

Map matching for low-sampling-rate GPS trajectories is an active research area, and different algorithms have been proposed. Lou et al. [2] proposed the ST-Matching algorithm for low-sampling-rate GPS trajectories, based on spatial-temporal analysis. The algorithm calculates observation probability, based on distance between GPS point and road segment, transition probability based on distance similarity and speed similarity. Then, a sequence of road segments, with the highest score, is identified as the map matching result. However, it is not reliable for filtering out incorrect candidate paths and determining the correct path, based on spatial and temporal information when the sampling rate exceeds 5 min. Hsueh et al. [3] extended the ST-Matching algorithm to STD-Matching algorithm by considering real-time direction of the GPS reading. The advantage of STD-Matching is that it can effectively filter out road segments with the wrong direction, which improves map matching accuracy, but it still confronts the same issue with STD-Matching when the sampling rate is very low. Quddus et al. [1] proposed a map matching method, based on weight-based shortest path, called stMM. Four different weights are assigned to each of the candidate links, including: (1) weight for perpendicular distance, (2) weight for bearing difference, (3) weight for shortest-path distance and (4) weight for heading difference. Then, the minimum weight path is calculated as the map matching result. Liu et al. [4] proposed a spatial and temporal conditional random field (ST-CRF) algorithm and considered consistency of driving direction. This algorithm calculates emission probability based on distance between GPS point and candidate point, transition probability by five influencing factors: (1) Distance between GPS point and candidate point, (2) shortest path distance and Euclidean distance between two GPS points, (3) average speed and speed constraint of road segment, (4) middle-point spatial distribution, and (5) driving direction. Dynamic programming was used to obtain the maximum probability path. Yin et al. [5] used length of the candidate route and road turning angle to estimate cost of the candidate route. Then, the authors computed the likelihood of a candidate path based on its cost and determined the path with the highest likelihood as the map matching result. In [1,5], they ignored vehicle speed and speed constraint of road segment, which may cause the final selected path to be unreachable under speed constraint. The above algorithms use a variety of features to handle map matching for low-sampling-rate GPS trajectories. Spatiotemporal features, including distance similarity, speed similarity and direction similarity, are used to filter out unreasonable candidate paths, reduce computation time and improve accuracy. However, when the sampling rate becomes very low, the uncertainty between consecutive GPS points becomes much larger, so it is very difficult to determine the correct path relying only on these features. 

Some other map matching methods take advantage of historical GPS trajectories with high sampling rates. Kai et al. [7] developed a map matching method, called HRIS (History-based Route Inference System). Historical high-sampling-rate GPS trajectories were first located on a road network to obtain historical routes with existing map matching techniques. Then, the low-sampling-rate trajectories were partitioned into a sequence of consecutive GPS location pairs, searched historical GPS trajectories through location pairs and popular routes were calculated between two locations. Next, HRIS connected consecutive local routes, based on a scoring function, which considered both the popularity of local routes and the confidence for connecting them. Finally, a dynamic programming algorithm was designed to calculate a global route with the highest score. Huang et al. [8] proposed a method to solve map matching for a low-sampling-rate GPS trajectory by mining frequent path patterns from historical high-sampling-rate GPS trajectories. This method performs a conventional map matching algorithm to obtain the paths of historical high-sampling-rate GPS trajectories. Next, the paths (and sub-paths) and their frequencies are stored in an FP (Frequent Pattern)-Forest index. Finally, the maximum probability path is calculated for a low-sampling-rate GPS trajectory based on the discovered frequent paths. The algorithms [7,8] above use additional historical high-sampling-rate GPS trajectory data to obtain frequent paths between any two locations in the road network because third-party historical GPS trajectory data can compensate for the uncertainty between consecutive GPS points with low sampling rates. However, these algorithms are susceptible to the quality of historical trajectory data, and drivers may not follow the frequent paths with rapid change of traffic conditions.

### 2.2. Trajectory Clustering Problem

Lee et al. [9] proposed a clustering algorithm for GPS trajectories. This algorithm partitioned the trajectory into sub-trajectories based on minimum description length (MDL) and computed perpendicular, parallel and angle distances to measure similarity between sub-trajectories. Smaller distance means higher similarity between sub-trajectories. Finally, it clustered the sub-trajectories based on DBSCAN algorithm [12]. Although the algorithm is efficient, it is only suitable for trajectory data without road constraints, such as hurricane movement trajectories and animal migration trajectories. Buchin et al. [10] also divided GPS trajectories into sub-trajectories. They measured the similarities based on Fréchet distance [13] and the discrete Fréchet distance between GPS sub-trajectories, respectively. However, computation cost for Fréchet distance is expensive. Additionally, this algorithm was designed for clustering GPS trajectories to detect commuting patterns in urban road networks, and it cannot be used for map matching. Kharrat et al. [11] proposed a trajectory clustering algorithm with road network constraint. This algorithm first computes the number of moving objects transiting from one road section to another, based on historical GPS trajectories. Next, it searches the densest road sections and utilizes them to generate dense paths on the road network. Last, it classifies the trajectories of moving objects into these dense paths. However, the performance of this algorithm relies on the traffic pattern discovered from additional trajectory datasets, and the movement of vehicles may not follow the discovered traffic pattern with the rapid development of road systems. None of these trajectory clustering methods work for map matching based on trajectory collaboration.

Many algorithms have been proposed to measure the similarity between GPS trajectories, which would be useful for trajectory clustering. For example, Mariescu-Istodor and Fränti [14] proposed a grid-based method to compute four different route measures - novelty, noteworthiness, similarity and inclusion. For similarity measurement, they first transformed route into cell representation and then utilized the Jaccard Index to measure the amount of similarity.

## 3. Methodology

### 3.1. Preliminaries

● Definition 1. Road network.

A road network is a directed graph, G=(V,E), where V is a set of vertices representing the terminal points of road segments, and E is a set of directed edges representing the road segments. Vertex $v_{i}\in V$ is a terminal point of road segments. Edge $e_{i}\in E$ is a road segment with a starting point $e_{j}.start$ and an end point $e_{j}.end$, where $e_{j}.start\in V$ and $e_{j}.end\in V$. 

● Definition 2. Path. 

A path is a sequence of consecutive road segments, denoted as $path=\left\{e_{1},e_{2},\cdots ,e_{m}\right\}$, where $e_{i}$ is a road segment with speed constraint and $e_{i}.end=e_{i+1}.start\left(1\leq i\leq m\right)$. The first and last vertex of path is denoted as path.first and path.last, and $path.first=e_{1}.start$ and $path.last=e_{m}.end$. 

**Figure 2:**
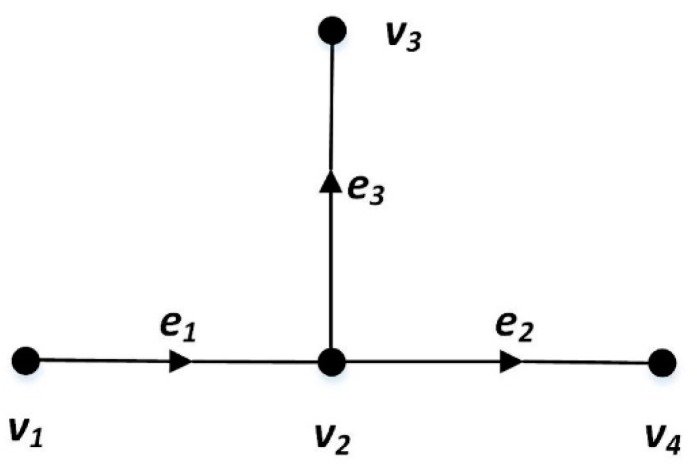
Example of Path.

Figure 2 gives an example of two paths. In Figure 2, two paths, $path_{1}$ and $path_{2}$, can be constructed by three road segments $e_{1},e_{2}$ and $e_{3}$. To be more specific, $path_{1}=\left\{e_{1},e_{2}\right\}$, and $path_{2}=\left\{e_{1},e_{3}\right\}$. In Figure 2, $path_{1}.first=v_{1}$ and $path_{1}.last=v_{4}$.

● Definition 3. GPS point. 

A GPS point p is a 4-tuple denoted as: p=(t,lat,lng,dir) where t is the timestamp of the GPS point, and lat, lng and dir are the latitude, longitude and direction of the location of the GPS point at time t.

● Definition 4. GPS trajectory. 

A GPS trajectory is a sequence of GPS points $trj=\left\{p_{1},p_{2},\cdots ,p_{m}\right\}$ where, $p_{i}.t-p_{i-1}.t>0,\;1\leq i\leq m$.

The map matching problem can be represented as follows: given a set of low-sampling-rate GPS trajectories, S, for each GPS trajectory trj∈S, this paper aims to locate trj onto the road network so that the path path of trj can be obtained.

### 3.2. Overview of Collaborative Map Matching

**Figure 3:**
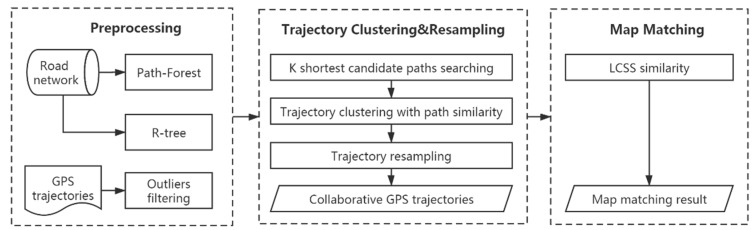
The framework of CMM algorithm.

In this paper, a collaborative map matching (CMM) algorithm is proposed to handle low-sampling-rate GPS trajectories. Figure 3 shows the framework of the CMM algorithm, which can be divided into three steps. The first is data preprocessing, where outliers are removed from GPS trajectories. To speed up the query operations on road segments and paths, an R-tree spatial index is built for road segment query, and a Path-Forest index is built to store paths in the road network. In the second step, k shortest distance paths [15] are first calculated out as candidate paths for each GPS trajectory. Then, the similarity between GPS trajectories is measured based on path similarity, and the DBSCAN algorithm is extended to cluster GPS trajectories. Next, a collaborative GPS trajectory with a high sampling rate is generated by resampling of a group of similar low-sampling-rate GPS trajectories in each cluster. In the last step, the LCSS similarity between the generated collaborative GPS trajectory and its candidate paths is calculated, and the path with the largest similarity is taken as the map matching result for all trajectories in the cluster. Each step will be discussed in detail.

### 3.3. Preprocessing

Data preprocessing is an important part of the proposed CMM algorithm. The first task is removing the outlier. If the difference between a GPS point and its nearest road segment exceeds the corresponding thresholds (including distance and direction), the GPS point will be taken as an outlier and removed. Second, an R-tree is built on road segments to facilitate the road segment query through spatial proximity. Finally, a Path-Forest index (Path-Forest) is built to store all paths between two road segments where path length is smaller than threshold $l_{\Delta }$, which could improve the efficiency of candidate route search in the road network. The Path-Forest is similar to the FP-Forest in [8], the difference being that FP-Forest is designed to store frequent paths (sub-paths) and their frequencies and Path-Forest is utilized to store all paths between any two road edges with length under $l_{\Delta }$. In a Path-Forest, each road segment in the road network will be taken as the root of a tree and the paths sourcing from the road segment will be stored in the tree. Each tree is associated with a hash table, where the key is the road segment ID and the value is a list storing the sequence of the corresponding road segments in the paths.

**Figure 4:**
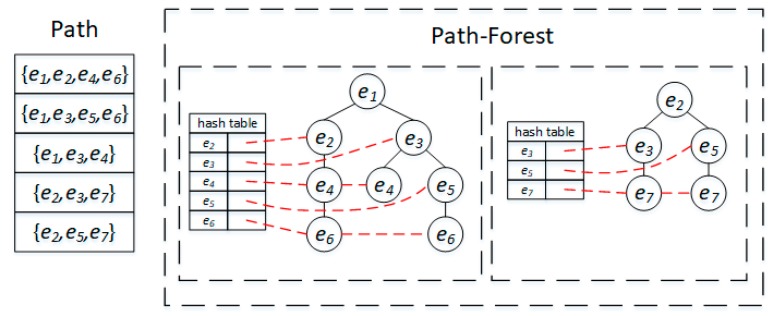
The Path-Forest.

It is efficient to retrieve all paths between two road segments in a Path-Forest. Figure 4 shows an example of path query from road segment $e_{1}$ to $e_{6}$. First, the tree with root $e_{1}$ and its associated hash table is searched out. In the hash table, the list pointing to $e_{6}$ will be identified. For each element in the list, the path from $e_{1}$ to $e_{6}$ will be obtained by tracing back, starting from $e_{6}$ in the tree. Thus, two paths from $e_{1}$ to $e_{6}$ will be searched out as $\left\{e_{1},\;e_{2},\;e_{4},\;e_{6}\right\}$ and $\left\{e_{1},\;e_{3},\;e_{5},\;e_{6}\right\}$.

### 3.4. Trajectory Clustering with Path Similarity

In this section, DBSCAN algorithm with path similarity is utilized for clustering low-sampling-rate GPS trajectories. The candidate paths are searched out for each low-sampling-rate GPS trajectory, and the similarity between GPS trajectories is measured based on the candidate paths. 

#### 3.4.1. Candidate Path Search

Given a search radius r, the candidate road segments for each GPS point can be queried out from an R-tree, and the paths to connect the candidate road segments of consecutive GPS points can be searched out from the Path-Forest. Unreasonable candidate paths should be pruned. In this paper, the candidate path pruning is implemented, based on a heading threshold β and an estimated temporal threshold, σ. The candidate path search with pruning method can be divided into two parts: candidate road segment query for each GPS point and path search in the Path-Forest with speed constraint.

1. Candidate Road Segment Query. When querying the surrounding road segments of a GPS point with radius, r(set as 100 m) in the R-tree, if the heading difference between a candidate road segment and the direction of the GPS point exceeds threshold β(set as 60°), the road segment will be dismissed from the query. Thus, candidate road segments with a similar direction for the GPS points could be searched.
2. Path Search. A complete candidate path of a GPS trajectory connects the candidate road segments from the first GPS point to the last GPS point, so it must search paths by connecting the candidate road segments of consecutive GPS points. However, the number of candidate paths for a GPS trajectory would be substantial in a road network. Thus, time constraint σ is calculated and used to filter out unreasonable paths and facilitate path search,
  (1) $$\sigma =\overset{m}{\underset{k=1}{\sum }}\frac{e_{k}.length}{e_{k}.speedlimit}$$

where σ is the calculated time threshold from GPS point $p_{i-1}$ to the next point $p_{i}$, and $e_{k}$ is a road segment on the candidate path from $p_{i-1}$ to $p_{i}$. Additionally, $e_{k}.speedlimit$ is the maximum speed allowed on $e_{k}$. Hence, threshold σ stands for the minimum time required to travel from $p_{i-1}$ to $p_{i}$. If the time interval $t_{i-1\to i}$ between $p_{i-1}$ and $p_{i}$ in Equation (2) is smaller than threshold σ, the path is considered unreasonable and is pruned.

(2) $$t_{i-1\to i}=p_{i}.t-p_{i-1}.t$$

Given a GPS trajectory $trj=\left\{p_{1},p_{2},\cdots ,p_{m}\right\}$, the pseudo-code of CPS is shown in Algorithm 1. The algorithm can be divided into two parts. The first part is candidate road segment query with radius r for each GPS point in lines 1–3. The set $CE_{i}$ is the set of candidate road segments of the GPS point $p_{i}$ in GPS trajectory trj. The second part is path search by connecting candidate road segments in lines 4–15. Path is the set of candidate road segment sequences for trj. As the algorithm starts from the candidate road segments of the first GPS point, in line 4, each candidate road segment in $CE_{1}$ is taken as a path and added into Path. From lines 5–15, the algorithm utilizes the Path-Forest to search candidate paths paths from $p_{i-1}$ to $p_{i}$, removing unreasonable paths through pruning and adding them into the candidate path set $Path{}^{\prime }$. Last, the previous candidate paths in Path are connected to the current paths in $Path{}^{\prime }$, and Path is updated with the result of connection.


**Algorithm 1: Candidate Path Search (CPS)**
**Input:** GPS trajectory $trj=\left\{p_{1},p_{2},\cdots ,p_{m}\right\}$; the set of edges in road network E; search radius r; heading change threshold β; **Output:** a set of $Path=\left\{path_{1},path_{2},\cdots ,path_{n}\right\}$1.**for** (i=1;i≤m;i++)2.  $CE_{i}\leftarrow$ CandidateRoadSegmentQuery($p_{i}$,E,r,β)3.
**end for**
4.
$Path\leftarrow CE_{1}$
5.**for** (i=2;i≤m;i++) 6.  $Path{}^{\prime }\leftarrow \emptyset$;7.  **foreach** (p in Path)8.   **foreach** (ce in $CE_{i}$)9.     $\sigma \leftarrow \sum_{k=1}^{m}e_{k}.length/e_{k}.speedlimit$
10.     paths← PathForestQuery(p.lastedge,ce,σ);11.     $Path^{\prime }\leftarrow paths$; 12.   **end foreach**13.  **end foreach**14.  $Path\leftarrow \mathrm{PathConnect}\left(Path,Path^{\prime }\right);$;15.
**end for**


#### 3.4.2. Trajectory Clustering

In order to supplement the missing information of low-sampling-rate GPS trajectories, similar GPS trajectories are clustered together. In this paper, the DBSCAN clustering method is extended for low-sampling-rate GPS trajectory clustering, based on path similarity instead of geometric similarity, as geometric similarity does not perform well for similarity measurements between low-sampling-rate GPS trajectories. The path similarity method measures similarity between low-sampling-rate GPS trajectories by comparing their candidate paths based on the Jaccard dissimilarity index. As the number of potential candidate paths for a GPS trajectory would be overwhelming, the k candidate paths with the shortest distance for each GPS trajectory are selected as candidate paths for path similarity measurement.

The Jaccard dissimilarity index $d\left(trj_{1},trj_{2}\right)$ between two GPS trajectories $trj_{1}$ and $trj_{2}$ is calculated based on the ratio of candidate paths whose dissimilarity is smaller than $\varepsilon_{p}$ to the total number of path combinations between two candidate sets. The calculation of $d\left(trj_{1},trj_{2}\right)$ is shown in Equation (3).

(3) $$d\left(trj_{1},trj_{2}\right)=1-\frac{\left|\pi \left(trj_{1}\right)\;\cap^{\text{​}}\pi \left(trj_{2}\right)\right|}{\left|\pi \left(trj_{1}\right)\;\cup^{\text{​}}\pi \left(trj_{2}\right)\right|}$$

$$\left|\pi \left(trj_{1}\right)\;\cap^{\text{​}}\pi \left(trj_{2}\right)\right|=\left\{d\left(path_{i},path_{j}\right)\langle \varepsilon_{p}\;|\;path_{i}\in \pi \left(trj_{1}\right),path_{j}\in \pi \left(trj_{2}\right)\right\}.count$$

$$\left|\pi \left(trj_{1}\right)\;\cup^{\text{​}}\pi \left(trj_{2}\right)\right|=\pi \left(trj_{1}\right).count\;*\;\pi \left(trj_{2}\right).count$$

where π(trj) denotes the set of candidate paths of the GPS trajectory trj, $\left|\pi \left(trj_{1}\right)\;\cap^{\text{​}}\pi \left(trj_{2}\right)\right|$ is the intersection of the candidate path sets of two trajectories and is composed of paths whose dissimilarity is smaller than $\varepsilon_{p}$. $\left|\pi \left(trj_{1}\right)\;\cup^{\text{​}}\pi \left(trj_{2}\right)\right|$ is the union of the two candidate path sets and is composed of all combinations between the candidate paths of the two trajectories.

The dissimilarity between two paths $path_{i}$ and $path_{j}$ is defined in Equation (4).

(4) $$d\left(path_{i},path_{j}\right)=1-\frac{2\times sim1\times sim2}{sim1+sim2}$$

$$sim_{1}=\frac{LCSS\left(path_{i},path_{j}\right).count}{path_{i}.count},sim_{2}=\frac{LCSS\left(path_{i},path_{j}\right).count}{path_{j}.count}$$

where $LCSS\left(path_{1},path_{2}\right)$ is the longest common subsequence [16] between two paths, and it calculates the number of common road segments in the two paths. For example, given two paths, $path_{1}=\left\{e_{1},e_{2},e_{3},e_{4},e_{5}\right\}$ and $path_{2}=\left\{e_{1},e_{3},e_{4},e_{6}\right\}$, the value $LCSS\left(path_{1},path_{2}\right)$ = $\left\{e_{1},e_{3},e_{4}\right\}$, $path_{1}.count=5$,$\;path_{2}.count=4$.

DBSCAN identifies clusters for spatial data based on density and requires two parameters: ε describing the maximum distance of a neighborhood, and MinPts describing the minimum number of points required to form a dense neighborhood. In this paper, the parameter ε is considered in two aspects, the maximum distance difference $\varepsilon_{l}$ between endpoints of trajectories, and the maximum dissimilarity $\varepsilon_{s}$ between two GPS trajectories based on path similarity. Thus, if the OD (origin-destination) pairs of GPS trajectories are distant from each other, they will be aggregated into different clusters.

The neighboring trajectories of each low-sampling-rate GPS trajectory could be obtained by Algorithm 2. As R-tree and Path-Forest have been built for GPS trajectories, it is efficient to retrieve the candidate paths around this trajectory (line 2). The dissimilarity measurement will filter out the GPS trajectories in which distance between endpoints is larger than threshold $\varepsilon_{l}$, and dissimilarity between two GPS trajectories is larger than threshold $\varepsilon_{s}$.


**Algorithm 2: Neighbor Query in DBSCAN**
**Input:** a set of GPS trajectory $trj=\left\{p_{1},p_{2},\cdots ,p_{m}\right\}$, parameters $\varepsilon_{l},\varepsilon_{s}$ **Output:** a set of $trjs=\left\{trj_{1},trj_{2},\cdots ,trj_{n}\right\}$1.trjs←∅;2.$trjs_{nei}\leftarrow$ rtree.query(trj.mbr);3.curPaths←trj.candidate;4.**foreach** (t in trjs_nei)5.  neiPaths←t.candidate;6.  **if** ($dis(t.p_{1},trj.p_{1}$)$<\varepsilon_{l}\;and\;dis(t.p_{n},trj.p_{m}$)$<\varepsilon_{l}$)7.   $d_{n}\leftarrow \mathrm{JaccardDissimilarity}\left(curPaths,neiPaths,\varepsilon_{s}\right)$
8.   **if** ($d_{n}<\varepsilon_{s}$)9.    trjs←t
10.   **end if**11.  **end if**12.
**end foreach**


Therefore, GPS trajectories can be clustered with the DBSCAN method with path similarity. Given certain values of MinTrjs, $\varepsilon_{l}$, $\varepsilon_{s}$, a GPS trajectory will be considered as a core trajectory if the number of GPS trajectories in its neighborhood exceeds the parameter MinTrjs. Then, a cluster can be generated based on the core trajectory with the conventional DBSCAN algorithm. If a GPS trajectory can be aggregated into more than one cluster, it will be assigned to the cluster with the largest number of GPS trajectories.

**Figure 5:**
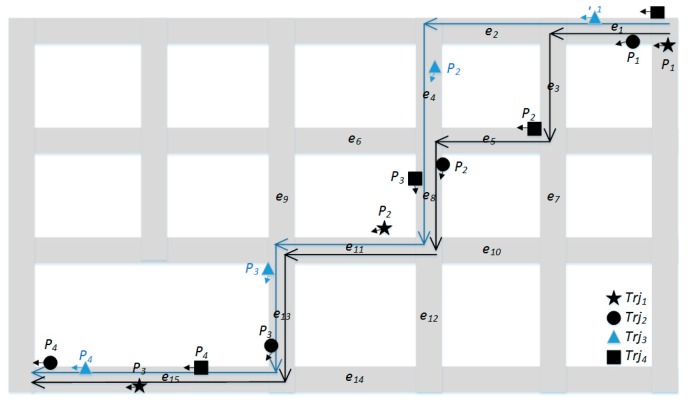
Clustering for low-sampling-rate GPS trajectories.

Figure 5 shows an example of trajectory clustering for four GPS trajectories (denoted in black and blue). The GPS trajectories $Trj_{1},Trj_{2},Trj_{4}$ are moving on the same path (black arrow), and the GPS trajectory $Trj_{3}$ is moving on another path (blue arrow). With $\varepsilon_{s}=0.3$ and MinTrjs=2 in the DBSCAN algorithm, the final clustering result for the four GPS trajectories is $Cluster_{1}=\left\{Trj_{1},Trj_{3},Trj_{4}\right\}$, $Noise=\left\{Trj_{2}\right\}$. Table 1, Table 2 and Table 3 show the road segment sequence of each path, the candidate paths of each GPS trajectory, and the dissimilarity between GPS trajectories in Figure 5, respectively.

### 3.5. Trajectory Resampling

GPS trajectories are aggregated into several groups with the DBSCAN clustering method. However, GPS trajectories in a cluster still confront the uncertainty issue due to low sampling rates. Therefore, for each cluster, it is necessary to integrate the low-sampling-rate GPS trajectories into a collaborative GPS trajectory, so they can supplement the missing information for each other. For example, the three GPS trajectories $Trj_{1},Trj_{2},Trj_{4}$ can supplement the missing information for each other in Figure 5. The generated collaborative GPS trajectory should integrate spatial information of low-sampling-rate GPS trajectories in the cluster for map matching. Thus, the trajectory resampling is different from averaging trajectory segment problems, which intends to obtain a representative trajectory for maintaining shape characteristics [17].

In this paper, a sliding window algorithm for trajectory resampling is proposed to generate collaborative GPS points from each trajectory cluster. The pseudocode of the algorithm is shown in Algorithm 3. At the beginning, the first and end points of all GPS trajectories in the cluster are collected into two sets of points, firstPs and endPs (line 2). Then, a GPS point is randomly selected from firstPs as the seed point, denoted as pSeed (line 3). A window composed of two circles, $C_{1}$ and $C_{2}$, centering at pSeed with radius $r_{s}$ and $2*r_{s}$ respectively, is generated (line 5). In the window, the GPS points inside $C_{1}$ can be retrieved quickly, and the center of the obtained GPS points is calculated as a new resampling GPS point, denoted as pNew (line 6–7). Next, a GPS point between $C_{1}$ and $C_{2}$ is randomly selected as a new seed point, pSeed. If there is no GPS point between $C_{1}$ and $C_{2}$, the radius $r_{s}$ is expanded to build a larger circle $C_{2}$ until a GPS point is obtained as the new seed point (line 8–9). Last, the window will slide forward until the end points in endPs are reached, and the sequence of generated sampling points can make up a collaborative GPS trajectory from the low-sampling-rate GPS trajectories in the cluster (line 4–11).


**Algorithm 3: Sliding-Window Algorithm for Trajectory Resampling**
**Input:** a set of GPS trajectories $trjs=\left\{trj_{1},trj_{2},\cdots ,trj_{n}\right\}$, search radius $r_{s}$ **Output:** a collaborative GPS trajectory $resultTrj=\left\{p_{1},p_{2},\cdots ,p_{m}\right\}$
1.resultTrj←∅; 2.firstPs←trjs.FirstPs, *endPs*←trjs.Last Ps;3.pSeed← random(firstPs);4.**while** ($pC_{1}C_{2}$.contains(*endPs*)) 5.  create $C_{1},C_{2}$ with radius $r_{s}$ and $2*r_{s}$;6.  $pC_{1}\leftarrow$ rtree.query(pSeed,$\;C_{1}$); 7.  pNew
$\leftarrow \;mean\left(pC_{1}\right)$; 8.  $pC_{1}C_{2}$
← rtree.query(pSeed,$C_{1}$,$C_{2}$); 9.  pSeed
←
$random\left(pC_{1}C_{2}\right)$;10.  resultTrj←pNew; 11.
**end while**


**Figure 6:**
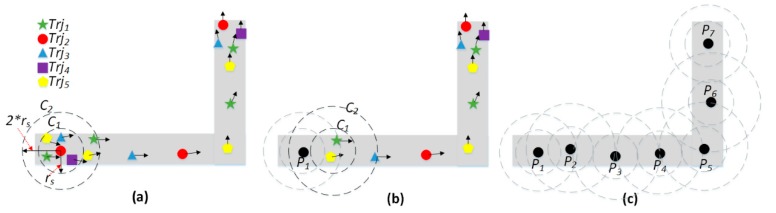
Trajectory cluster resampling. (**a**) Seed selection and window generation. (**b**) Point resampling and window slide. (**c**) Collaborative trajectory generation.

Figure 6 shows the procedure of trajectory resampling in a cluster. In this figure, five trajectories (green, red, blue, purple and yellow) are grouped into a cluster; the small black arrow is the real-time heading of a GPS point. In Figure 6a, the red GPS point is randomly selected as the seed point, and two circles $C_{1}$ and $C_{2}$ are generated, centering as the seed point. In $C_{1}$, five GPS points are retrieved, and the center point is created as a new resampling point and denoted as $P_{1}$ in Figure 6b. In Figure 6a, two GPS points exist between $C_{1}$ and $C_{2}$, and the next seed point will be randomly selected from them. As the window reaches the end GPS points of the cluster, a collaborative GPS trajectory is generated and composed from $P_{1}$ to $P_{7}$, as shown in Figure 6c.

### 3.6. Map Matching for the Supplemented Trajectory

After trajectory resampling, a collaborative GPS trajectory is generated from each cluster, and it will be matched to one of the candidate paths. Given a GPS trajectory $trj=\left\{p_{1},p_{2},\cdots ,p_{m}\right\}$ and a candidate path $path=\left\{e_{1},e_{2},\cdots ,e_{n}\right\}$, LCSS [18] is utilized to measure the similarity between trajectory and path. The path with the largest LCSS value will be taken as the map matching result for all low-sampling-rate GPS trajectories in the corresponding cluster:

(5) $$\mathrm{Sim}\left(p_{i},e_{j}\right)=\{\begin{array}{cc} 0, & Dist\left(p_{i},e_{j}\right)>\varepsilon_{d}\\ 1-\frac{Dist\left(p_{i},e_{j}\right)}{\varepsilon_{d}} & otherwise \end{array}.$$

$Dist\left(p_{i},e_{j}\right)$ is the Euclidean distance from a GPS point $p_{i}$ to the closest point on the road segment $e_{j}$. If $Dist\left(p_{i},e_{j}\right)>\varepsilon_{d}$, this GPS point $p_{i}$ cannot be matched to this road segment $e_{j}$.The subsequence of a trajectory trj from its first GPS point to the i-th GPS point is defined as $trj\left(i\right)=\left\{p_{1},p_{2},\ldots ,p_{i}\right\},\;1\leq i\leq m$ and the subsequence of path from the first road segment to the j-th road segment as $path\left(j\right)=\left\{e_{1},e_{2},\ldots ,e_{j}\right\},\;1\leq j\leq n$. The LCSS value, denoted as L(trj(i), path(j)), between trj(i) and path(j) is defined as,

(6) $$L\left(trj\left(i\right),\;path\left(j\right)\right)=max\{\;L\left(trj\left(i\right),\;path\left(j-1\right)\right),L\left(trj\left(i-1\right),\;path\left(j\right)\right)+Sim\left(p_{i},\;e_{j}\right)\}$$

With Equation (6), a dynamic programming algorithm could be used to calculate the LCSS distance between trj and path, and the candidate path with the largest LCSS value is selected as the map matching result.

## 4. Experiment

### 4.1. Study Area

**Figure 7:**
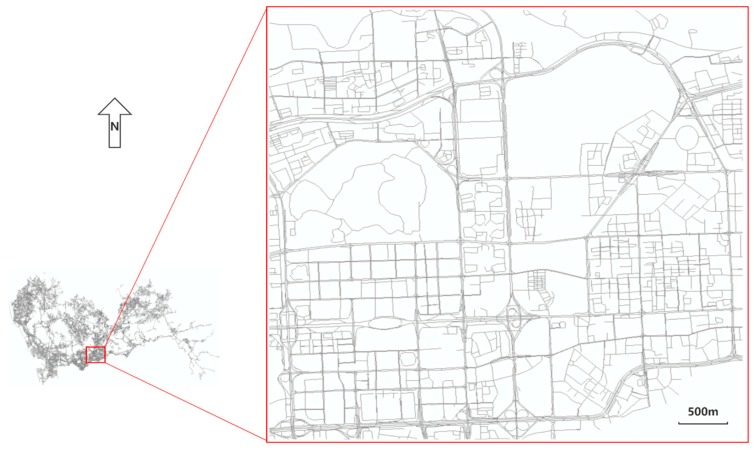
Study area of Shenzhen map.

In this section, the performance of the proposed CMM method was evaluated using real and simulated GPS trajectory datasets in a study area (22.532° N–22.576° N and 114.052° E–114.103° E) of Shenzhen, China. The method was implemented in C#. The experiments were conducted on a 3.6 GHz Core i7 PC with 16GB of RAM. The road network in the selected region contains 7007 road vertices and 7949 road segments, which is shown in Figure 7.

### 4.2. Dataset

In this paper, experiments were conducted to evaluate the performance of the proposed CMM algorithm on both, a real and a simulated GPS trajectory dataset.

#### 4.2.1. Real GPS Trajectory Dataset

The real taxi GPS trajectory dataset [19], containing more than 46 million GPS points, was collected in Shenzhen, China. The format of GPS points, includes the taxi ID, longitude, latitude, timestamp, speed and occupancy status. The sampling rate of the GPS trajectories is approximately 15 s. The trajectory data was displayed on the map, and 100 GPS trajectories were selected for validation. The 100 GPS trajectories were selected by visual inspection so that they are diverse in length and located at different locations in the study area. The 100 GPS trajectories are manually labelled for ground truth. To obtain low-sampling-rate GPS trajectories, each GPS trajectory is sampled multiple times with the sampling rate of 3 min and different starting points. In total, 734 GPS trajectories with sampling rate around 3 min were generated. The instantaneous direction of GPS points of the generated trajectory is also manually labelled, as the direction information is unavailable in this real GPS trajectory dataset.

**Figure 8:**
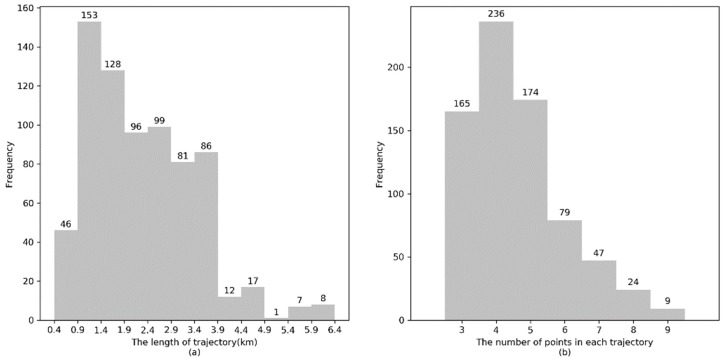
Distribution of real GPS trajectories. (**a**) Trajectory length. (**b**) Number of points in each trajectory.

Figure 8a shows the length of the majority of GPS trajectories in the real dataset is between 1 km and 4 km, Figure 8b shows that the number of GPS points in each trajectory is approximately 3 to 5.

#### 4.2.2. Trajectory Simulation

**Figure 9:**
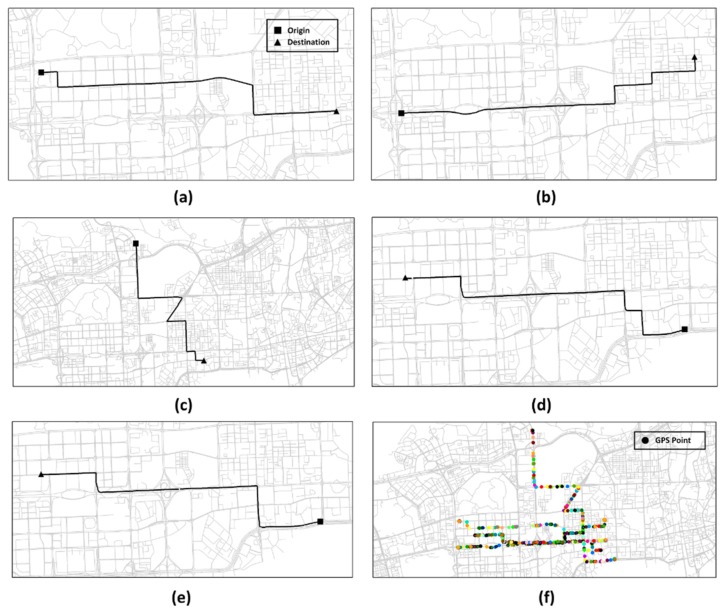
Spatial distribution of selected road paths and simulated GPS trajectories. (**a**,**b**) Road paths under scenario $T_{1}$. (**c**) Road path under scenario $T_{2}$. (**d**,**e**) Road paths under scenario $T_{3}$. (**f**) Spatial distribution of simulated GPS trajectories.

In this paper, GPS trajectories were simulated to investigate the performance of map matching methods in two aspects. First, the performance of map matching methods versus different sampling rates (from 2 min to 10 min) was studied, based on simulated trajectory data, which is difficult to obtain from a real GPS trajectory dataset. Second, GPS trajectories were simulated to study the performance of map matching methods under three different scenarios $T_{1}$, $T_{2}$ and $T_{3}$. $T_{1}$ represents GPS trajectories moving along a path with multiple turns. $T_{2}$ represents GPS trajectories moving along a path with a large detour. $T_{3}$ represents GPS trajectories moving along two separate paths which vary slightly at a short sub-path. Figure 8 illustrates the road paths where GPS trajectories are simulated. Figure 9a,b represent scenario $T_{1}$; Figure 9c represents scenario $T_{2}$; and Figure 9d,e represent scenario $T_{3}$. In Figure 9f, 50 GPS trajectories were simulated on each road path, and the simulated GPS trajectories are shown in different colors.

Simulated GPS points are associated with a set of attributes including trip ID, time, longitude, latitude, direction. To simulate a GPS trajectory on path $path=\left\{e_{1},\;e_{2},\ldots ,\;e_{m}\right\}$, the velocity of the vehicle moving on the path is first estimated using Equation (7):

(7) $$\alpha =\sum_{k=1}^{m}\left(e_{k}.speedlimit*\frac{e_{k}.length}{path.length}\right).$$

The weighted average maximum speed limit α (m/s) on the path is calculated as the velocity of the simulated GPS trajectory. $e_{k}.speedlimit$ and $e_{k}.length$ are the speed limit and the length of the road segment $e_{k}$, respectively. path.length is the total length of the path. The procedure of GPS trajectory simulation is as follows:

1. Simulate the first point $p_{1}$ based on the starting node of the path, and is denoted as $p_{prev}.$
2. Estimate the location of the next GPS point $p_{next}$. First, a random velocity ν=random(0,α) is generated for the vehicle. Given the sampling rate, the travelled distance from $p_{prev}$ to $p_{next}$ along the path can be calculated as ν∗t. Thus, the location of $p_{next}$ on the path can be obtained and denoted as ($p_{next}.x,\;p_{next}.y$). Location bias $x_{bias}\sim N\left(0,40\right)$ and $\;y_{bias}\sim N\left(0,40\right)$ is added to simulate the position deviation of the vehicle away from the road. Thus, the location of $p_{next}$ is simulated as $\left(p_{next}.x+x_{bias},\;p_{next}.y+y_{bias}\right)$.
3. Simulate the direction of $p_{next}$. With the location of $p_{next}$, the road segment where $p_{next}$ is located is identified and the direction of the road segment is denoted as $direction_{road}$. Then, a random $direction_{bias}=random\left(-30,30\right)$ is generated as the heading bias of $p_{next}.$ Thus, the direction of $p_{next}$ can be simulated as $direction_{road}+direction_{bias}.$
4. Mark $p_{next}$ as $p_{prev}$, and repeat steps 1–3 to simulate the next GPS point until the vehicle reaches the end node along the path. Set a unique trajectory ID for the simulated GPS trajectory.

**Figure 10:**
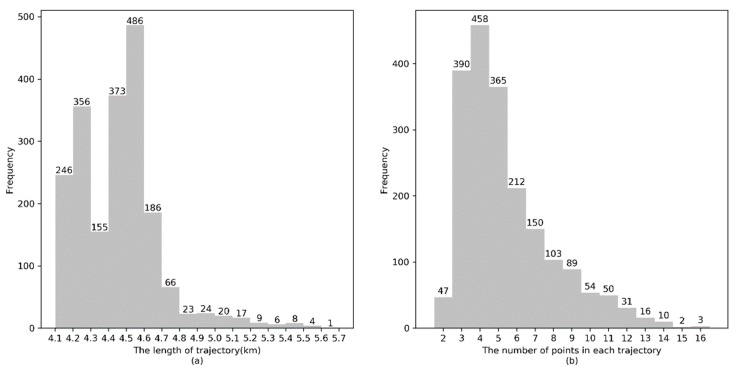
Distribution of simulated GPS trajectories. (**a**) Trajectory length. (**b**) Number of points in each trajectory.

The length distribution of simulated GPS trajectories and the number of GPS points in each simulated GPS trajectory are illustrated in Figure 10a, and Figure 10b, respectively. It can be observed that the length of the majority of simulated GPS trajectories is approximately 4.5 km and the number of GPS points in a trajectory is approximately 3 to 5.

### 4.3. Parameter Configuration

In this section, the performance of CMM is compared with two baseline methods, ST-Matching [2] and STD-Matching [3]. The preprocessing step is the same for all three methods, including outlier removal, the construction of R-tree and Path-Forest. The configuration of CMM is set in Table 4. The experiments are conducted based on the configuration without specific statement.

Two metrics, precision by length of road segments and recall by length of road segments, are used to evaluate the effectiveness of the map matching methods, and they are defined as follows:

$$Precision=\frac{Length\;of\;correctly\;matched\;road\;segments}{Length\;of\;predicted\;path}$$

$$Recall=\frac{Length\;of\;correctly\;matched\;road\;segments}{Length\;of\;ground\;true\;path\;}$$

### 4.4. Performance Evaluation on Real Trajectory Dataset

In this paper, the performance of the proposed CMM method and two baseline methods was investigated using a real GPS trajectory dataset. First, the effectiveness and efficiency of the three methods were compared in terms of precision, recall and running time. Second, parameter tuning was conducted to investigate the impact of two significant parameters on the CMM method. Third, the effectiveness of path similarity was studied and compared with the LCSS and Edit Distance on Real Sequence (EDR) methods.

#### 4.4.1. Overall Performance Evaluation

This section investigates precision, recall and running time of the CMM method, ST-Matching method and STD-Matching method. The average running time per GPS point of the three methods are shown in Table 5. It can be observed that the average running time per GPS point of CMM is approximately 2.97 milliseconds (ms), while the average running time per GPS point for ST-Matching and STD-Matching method are 29.60 ms, and 28.37 ms, respectively. CMM is much more efficient than ST-Matching and STD-Matching because once the trajectories are clustered in the same cluster, CMM conducts map matching for all GPS trajectories belonging to the cluster, while the other two methods process the GPS trajectory individually. 

The precision and recall of the three map matching methods are shown in Table 6. The precision and recall of CMM are 0.865 and 0.872 for real GPS trajectories with the sampling rate of around 3 min, which performs better than both ST-Matching and STD-Matching. ST-Matching has the smallest precision and recall because it could not distinguish the correct path effectively based on the spatial and temporal features. STD-Matching involves direction information and has a better performance than ST-Matching. The CMM method groups similar GPS trajectories together and solves the uncertainty issue by generating a new collaborative trajectory with information supplementation of the low-sampling-rate GPS trajectories, which demonstrates that CMM has the best performance out of the three methods in terms of precision and recall.

#### 4.4.2. Performance Evaluation for Path Similarity

This section evaluates the performance of the proposed path similarity method. Understanding how the similarity measure is affected by sampling rates was investigated. The similarity between the trajectory with varied sampling rates and the origin trajectory is expected to be 100%.

**Figure 11:**
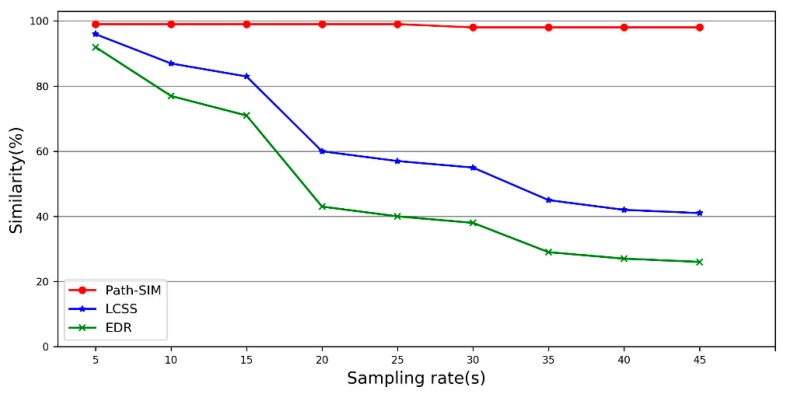
Performance evaluation for path similarity (Path-SIM), LCSS and EDR.

The performance of the path similarity method was compared with two widely used methods, Longest Common Subsequence (LCSS) [16] and Edit Distance on Real Sequence [20] methods. LCSS and EDR are count-based methods. LCSS counts the number of matched pairs, and EDR counts the cost of operations needed to fix the unmatched pairs. The sampling rates could have a great impact on the distance calculation between the two trajectories, especially the GPS trajectories with a low sampling rate. However, the count-based similarity measurement methods are less sensitive to sampling rates. To be more specific, regardless of distance between the two consecutive GPS points, count-based similarity measurement methods do not change the value. The experiment results are shown in Figure 11.

It can be observed in Figure 11 that path similarity method (Path-SIM) has better performance than LCSS and EDR methods. Path-SIM is almost not affected by sampling rates because the candidate paths for a trajectory have a small change with the varied sampling rates. However, LCSS and EDR are more sensitive to the change of sampling rates compared to Path-SIM.

#### 4.4.3. Parameter Tuning

CMM has two important parameters - the k value for the top k shortest distance paths, and the dissimilarity threshold $\varepsilon_{s}$ for defining neighborhood in the extended DBSCAN. This section investigates the impact of two parameters, k and $\varepsilon_{s}$ on the performance of CMM, based on real GPS trajectories. In this experiment, k is set from 1 to 15, and $\varepsilon_{s}$ is 0.1 to 0.8.

**Figure 12:**
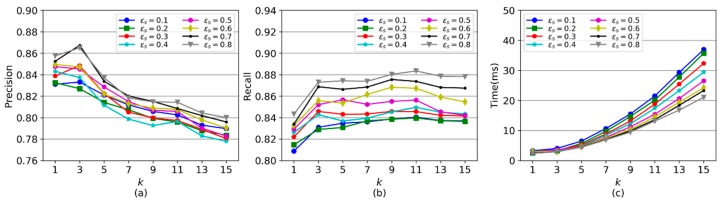
The performance of CMM with different values of k and $\varepsilon_{s}$. (**a**) Precision. (**b**) Recall. (**c**) Running time.

Figure 12a,b display the precision and recall of map matching with different k and $\varepsilon_{s}$ values. First, when $\varepsilon_{s}$ is fixed, the precision and recall increase as k grows from 1 to 3. When k is set as 1, similar GPS trajectories cannot be well clustered together using path similarity because the shortest distance path is usually not the real path for some GPS trajectories. Thus, GPS trajectories in a cluster may not be enough to supplement information for each other, which results in precision and recall being smaller than when k is set as 3. With k increasing from 3 to 15, the precision decreases gradually, while the recall stays steady. This arises because a greater k will result in a larger number of unreasonable candidate paths with detours, which would compromise the precision of CMM but has a small impact on the recall of CMM. Second, when k is fixed, the precision and recall increase with the growth of $\varepsilon_{s}$ because more similar GPS trajectories can be aggregated with a larger $\varepsilon_{s}$, which leads to more spatial information of similar GPS trajectories being incorporated together for map matching.

Figure 12c shows the running time of CMM rises with the growth of k and declines with the growth of $\varepsilon_{s}$. With a greater k, more candidate paths are searched out for GPS trajectories, which increases the computation time for trajectory similarity measurements in trajectory clustering. A smaller $\varepsilon_{s}$ value causes a smaller number of GPS trajectories in one cluster and increases the total number of clusters. Therefore, CMM requires more running time to conduct map matching, as shown in the figure.

### 4.5. Performance Evaluation on Simulated Trajectory Dataset

In this paper, the performances of CMM, ST-Matching and STD-Matching were also investigated on a simulated GPS trajectory dataset. First, the impact of sampling rate on the performance of the three map matching methods was investigated. Second, the three map matching methods were studied under different scenarios. Third, the impact of trajectory data size on the performance of the three map matching methods was studied.

#### 4.5.1. Performance versus Sampling Rate

**Figure 13:**
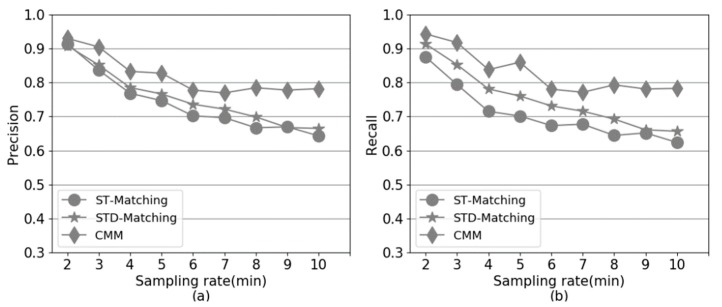
The performance comparison versus different sampling rates. (**a**) Precision. (**b**) Recall.

Figure 13 shows the performance of CMM, ST-Matching and STD-Matching, with different sampling rates, on simulated GPS trajectories. It can be observed that CMM always performs better than ST-Matching and STD-Matching when GPS sampling rate varies from 2 min to 10 min. Compared to ST-Matching and STD-Matching, the precision and recall of CMM are much less sensitive to the sampling rates. When the sampling rate is very low, e.g., 8 min, consecutive GPS points become more distant from each other, resulting in large uncertainty for the real path of the trajectory. Thus, the precision and recall of ST-Matching and STD-Matching drop significantly based on spatial and temporal features. CMM supplements the spatial information of low-sampling-rate GPS trajectories with trajectory collaboration, which can alleviate the larger uncertainty issue caused by lower sampling rates.

#### 4.5.2. Performance Evaluation under Different Scenarios

**Figure 14:**
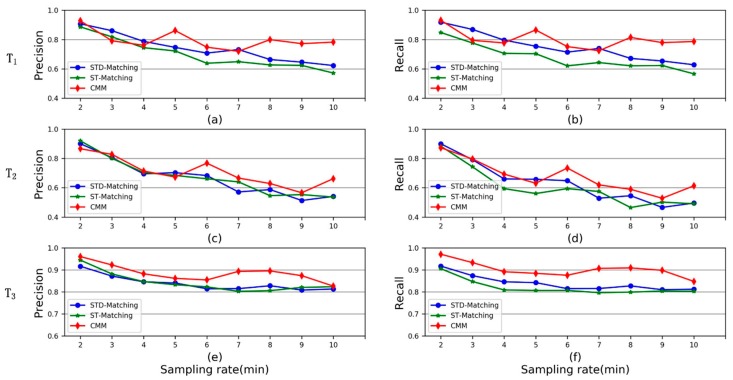
The performance comparison of CMM, ST-Matching and STD-Matching under different scenarios. (**a**,**b**) Scenario $T_{1}$. (**c**,**d**) Scenario $T_{2}$. (**e**,**f**) Scenario $T_{3}$.

The performance of the three map matching methods was investigated under three scenarios $T_{1}$, $T_{2}$ and $T_{3}$. The experiment results are displayed in Figure 14. It can be observed in Figure 14a–d that CMM performs better than ST-Matching and STD-Matching under scenarios $T_{1}$ (multiple turns) and $T_{2}$ (detour). For scenario $T_{3}$, the precision and recall of CMM, ST-Matching and STD-Matching are all over 0.8. It can be concluded that CMM can effectively aggregate low-sampling-rate GPS trajectories into different clusters based on path similarity, if two groups of trajectories move along distinct road paths.

**Figure 15:**
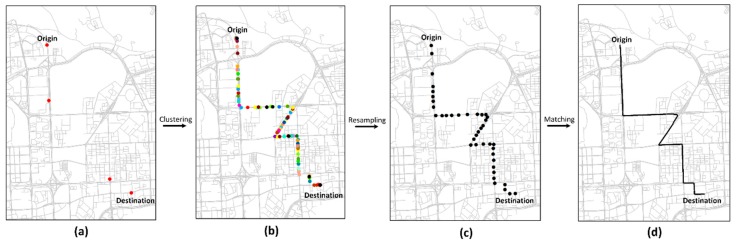
Visualization of map matching for a simulated low-sampling-rate GPS trajectory. (**a**) Low-sampling rate GPS trajectory. (**b**) Trajectory cluster. (**c**) Collaborative trajectory. (**d**) Map matching result.

Figure 15 visualizes the map matching results for a simulated GPS trajectory under $T_{2}$. Figure 15a shows that the sampling rate of the simulated GPS trajectory is very low, and it is very difficult to identify the real path of the trajectory. Figure 15b shows the cluster where the low-sampling-rate GPS trajectory is aggregated with the path similarity method. Figure 15c represents the collaborative trajectory of the cluster after resampling, and Figure 15d shows the map matching result of the collaborative trajectory.

**Figure 16:**
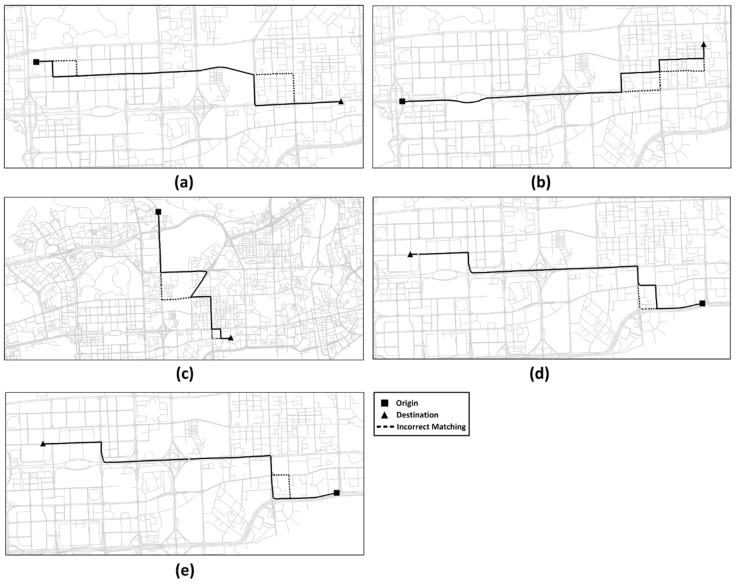
Incorrect map matching parts of ST-Matching algorithm. (**a**,**b**) Scenario $T_{1}$. (**c**) Scenario $T_{2}$. (**d**,**e**) Scenario $T_{3}$.

Figure 16 illustrates the incorrect paths (shown in the dashed lines) identified by the ST-Matching algorithm under different scenarios. It can be observed in the figure that incorrect map matching components are a result of the correct candidate path not being distinguished from other candidate paths, based on spatial and temporal features, if consecutive GPS points are far from each other.

#### 4.5.3. CMM Performance versus Trajectory Data Size

This section evaluates the impact of trajectory data size on the performance of CMM. The performance of CMM is studied with a varied number of simulated GPS trajectories (50, 300, 500, 700, 1000) on the paths. 

**Figure 17:**
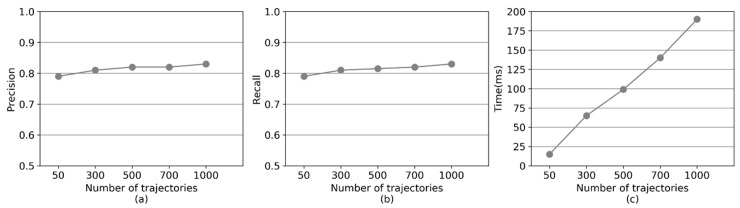
The performance of CMM with different numbers of GPS trajectories. (**a**) Precision. (**b**) Recall. (**c**) Running time.

In Figure 17a,b, the precision and recall of CMM grow with an increase in trajectory data size. A greater GPS trajectory data size, results in further GPS trajectories used to make up for the missing information. This, in turn, leads to the generation of a collaborative GPS trajectory containing more information after resampling, and thereby, reducing the uncertainty of the group of low-sampling-rate GPS trajectories.

Figure 17c shows the running time of CMM with different sizes of GPS trajectory data. The average running time per GPS point grows linear with increased trajectory data size. This is because the running time of CMM is mainly composed of two parts—trajectory similarity calculation and candidate path search. With Path-Forest, the candidate path search is efficient. Thus, the time cost for candidate path search does not grow, even if the size of trajectory data is greatly expanded. However, the running time for trajectory similarity calculation will increase with the growing number of GPS trajectories. 

## 5. Conclusions and Future Work

In this paper, a collaborative map matching method (CMM) was proposed for low-sampling-rate GPS trajectories. CMM first groups similar GPS trajectories into clusters based on path similarity and then supplements the missing information of GPS trajectories by resampling. A collaborative trajectory is then extracted for each cluster and is matched to the road network. Experiments were conducted in a real, and a simulated, GPS trajectory dataset. The experiment results show that the proposed CMM outperforms the baseline method in both, effectiveness and efficiency.

In CMM, the similarity measurement for low-sampling-rate GPS trajectory is considered, based on path similarity, which overcomes the deficiency of geometry similarity. A trajectory resampling algorithm is proposed to extract a high-sampling-rate GPS trajectory from each cluster of low-sampling-rate GPS trajectories, which could effectively reduce the uncertainty issue of map matching.

This work can be improved in the following ways: First, the current path similarity method only considers the number of common road segments when measuring the similarity between candidate paths. In future, more features can be involved for path similarity measurement. For instance, the average moving speed of GPS trajectories on candidate paths can be considered for path similarity measurement. Additionally, the grid-based method C-SIM was proposed and seems promising for measuring similarity between the trajectories [14]. The method uses only the spatial aspect of routes and ignores the order of traveling, which makes it simple and fast. It converts the GPS points to cells and fills the gap between cells using linear interpolation if two consecutively generated cells are not adjacent. However, the size of the grid is crucial and should be carefully determined for low sampling trajectories. For this study, if the grid size is too large, dissimilar GPS trajectories would be aggregated into one group; if the grid size is too small, similar GPS trajectories may not be effectively clustered into one group. Therefore, how to utilize the grid-based method in map matching for a low-sampling-rate trajectory will be investigated in future. Second, for each trajectory cluster, only location information of GPS points was considered for trajectory resampling. However, the speed and heading information of new generated GPS points should be estimated after resampling, which could produce a better map matching result. The CMM method will be deployed on a distributed computing platform to handle massive low-sampling-rate GPS trajectories throughout the city.

## Figures and Tables

**Table 1:** Paths and the corresponding road segment sequences.

Path ID	Road Segment Sequence in the Path
$path_{1}$	$\left\{e_{1},e_{2},e_{4},e_{8},e_{11},e_{13},e_{15}\right\}$
$path_{2}$	$\left\{e_{1},e_{3},e_{5},e_{8},e_{11},e_{13},e_{15}\right\}$
$path_{3}$	$\left\{e_{1},e_{3},e_{7},e_{10},e_{11},e_{13},e_{15}\right\}$
$path_{4}$	$\left\{e_{1},e_{2},e_{4},e_{6},e_{9},e_{13},e_{15}\right\}$
$path_{5}$	$\left\{e_{1},e_{3},e_{5},e_{8},e_{12},e_{14},e_{15}\right\}$

**Table 2:** Trajectories and the corresponding candidate paths.

Trajectory ID	Candidate Paths
$Trj_{1}$	$path_{1},\;path_{2},\;path_{3}$
$Trj_{2}$	$path_{1},\;path_{2}$
$Trj_{3}$	$path_{1},\;path_{4}$
$Trj_{4}$	$path_{2},\;path_{5}$

**Table 3:** Dissimilarity between trajectories.

$d\left(Trj_{i},Trj_{j}\right)$	$Trj_{1}$	$Trj_{2}$	$Trj_{3}$	$Trj_{4}$
$Trj_{1}$	-	0.16	0.5	0.33
$Trj_{2}$	0.16	-	0.25	0.25
$Trj_{3}$	0.5	0.25	-	0.75
$Trj_{4}$	0.33	0.25	0.75	-

**Table 4:** Configuration of CMM.

Parameters		
Path distance threshold in Path-Forest	$l_{\Delta }$	6 km
k candidate path for path similarity measurement	k	3
Distance threshold in trajectory clustering	$\varepsilon_{l}$	50 m
Dissimilarity threshold in trajectory clustering	$\varepsilon_{s}$	0.8
MinTrjs in trajectory clustering	MinTrjs	5
Trajectory-Resampling radius	$r_{s}$	50 m
LCSS distance threshold	$\varepsilon_{d}$	100 m

**Table 5:** Average Running Time per GPS Point on Real GPS Trajectory Dataset.

Method	Time (ms)
ST-Matching	29.60
STD-Matching	28.37
CMM	**2.97**

**Table 6:** Precision and Recall on Real GPS Trajectory Dataset.

Method	Precision	Recall
ST-Matching	0.817	0.721
STD-Matching	0.855	0.792
CMM	**0.865**	**0.872**
